# Systematic review of the significance of neutrophil to lymphocyte ratio in anastomotic leak after gastrointestinal surgeries

**DOI:** 10.1186/s12893-023-02292-0

**Published:** 2024-01-06

**Authors:** Sarvin Es Haghi, Monireh Khanzadeh, Shirin Sarejloo, Fariba Mirakhori, Jairo Hernandez, Emma Dioso, Michael Goutnik, Brandon Lucke-Wold, Arshin Ghaedi, Shokoufeh Khanzadeh

**Affiliations:** 1 Faculty of Medicine, Shahid Beheshti University of Medical Sciences, Tehran, Iran; 2 Geriatric & Gerontology Department, Medical School, Tehran University of medical and health sciences, Tehran, Iran; 3 Cardiovascular Research Center, Shiraz University of Medical Sciences, Shiraz, Iran; 4 Zanjan University of Medical Science, Zanjan, Iran; 5 University of Florida, Gainesville, USA; 6 University of Utah, Salt Lake City, UT USA; 7 University of Florida, Gainesville, USA; 8 Department of Neurosurgery, University of Florida, Gainesville, USA; 9 Student Research Committee, School of Medicine, Shiraz University of Medical Sciences, Shiraz, Iran; 10 Trauma Research Center, Shahid Rajaee (Emtiaz) Trauma Hospital, Shiraz University of Medical Sciences, Shiraz, Iran; 11 Tabriz University of Medical Sciences, Tabriz, Iran

**Keywords:** Anastomotic leak, Surgery, Neutrophil to lymphocyte ratio, Meta-analysis

## Abstract

**Introduction:**

The inflammatory response is thought to be a critical initiator of epigenetic alterations. The neutrophil to lymphocyte ratio (NLR), a biomarker of inflammation, is computed by dividing the number of neutrophils by the number of lymphocytes. The primary goal of this systematic review and meta-analysis was to evaluate the pre-operative NLR of gastrointestinal surgery patients who had an anastomotic leak (AL) in comparison to those who did not AL.

**Methods:**

We performed a comprehensive search for relevant papers published before May 4, 2022, using PubMed, Scopus, and Web of Science. Standardized mean difference (SMD) with a 95% confidence interval (CI) was pooled in meta-analysis to yield a summary estimate. We utilized the random-effects model to create pooled effects since we discovered a substantial heterogeneity level. For evaluating quality, the Newcastle-Ottawa scale (NOS) was implemented.

**Results:**

The research comprised 12 studies with a total of 2940 individuals who had GI operations, 353 of whom went on to develop AL. We discovered that patients who had GI surgeries and acquired AL had significantly higher NLR levels than those who did not (random-effects model: SMD = 0.75, 95% CI = 0.11–1.38, *p* = 0.02). Patients with AL showed significantly higher NLR levels than control group in retrospective studies (SMD = 0.93, 95% CI = 0.20–1.66, p=0.01) but not in prospective studies (SMD = − 0.11, 95% CI = − 0.65–0.43, *p* = 0.69), according to the subgroup analysis based on research design. Subgroup analysis based on ethnicity yielded that white patients with AL exhibited significantly higher NLR values than the control group (SMD = 1.35, 95% CI = 0.01–2.68, *p* = 0.04) but this result was not applied to East Asian patients (SMD = 0.14, 95% CI = -0.13–0.41, *p* = 0.29).

**Conclusion:**

Our research suggests a potential association between preoperative NLR and postoperative AL. However, it is essential to acknowledge the variability in the findings, with significantly higher NLR levels observed in retrospective studies and among white patients, but not consistently replicated in prospective studies and among East Asian patients. Further investigations with larger and more diverse cohorts are warranted to validate these findings and explore potential factors contributing to the observed discrepancies.

## Introduction

Epigenetic alterations are thought to be considerably triggered by the inflammatory response. In a number of surgical operations, the neutrophil to lymphocyte ratio (NLR) has been suggested as an inflammatory measure and predictive tool. Recent investigations have demonstrated that NLR is a more accurate predictor of patient survival than neutrophil or lymphocyte counts alone [1]. The significance of this ratio as an indicator for other outcomes, particularly anastomotic leakage (AL), has also been underlined by recent data from the literature on the gastrointestinal (GI) system [2]. The overall prognosis of these patients is worsened by AL following gastric resections, with substantial morbidity and death rates that might exceed 60% [3–5]. Additionally, AL is linked to increased short-term mortality and higher healthcare system expenses [6–11]. A systematic review is necessary to inform clinical decision-making since recent studies on this subject are constantly being published. The key to helping doctors implement early treatments and enhance outcomes is knowing what an increased ratio would signify for a patient following GI surgery. Some studies have shown a significant correlation between NLR and AL in a variety of GI surgeries, but some other do not. To our knowledge, there are no systematic reviews of the literature that are currently accessible that address the applicability of NLR in this context. In order to guide clinical management and enhance outcomes, this systematic review and meta-analysis set out to compile the information that is currently available on the function of the NLR in predicting AL in GI surgery. This is the first research in this field that we are aware of.

## Material and method

We followed the Preferred Reporting Items for Systematic Reviews and Meta-Analyses guidelines for conducting our systematic review and meta-analysis [12]. This study was registered in PROSPERO (ID: 387732).

### Data sources and searches

On May 4, 2022, an electronic search of the three major databases Scopus, Web of Science, and PubMed, was carried out. ((neutrophil AND lymphocyte AND ratio) OR NLR) AND (“anastomo*”) AND (“leak*” OR “dehiscence”) were the search terms that we used. To discover other relevant papers, reference lists of included articles were examined. In addition, a free-text search of the OpenGrey grey literature repository was conducted.

### Study selection

To guarantee a systematic search of the current literature, we select eligible studies using the PICOS (population, intervention, control, outcomes, and study design) principle. The following paragraph is the criteria used for inclusion:

- Population: Patients undergoing GI surgeries who developed AL.
- Intervention. NLR.
- Control. Patients undergoing GI surgeries who did not develop AL.
- Outcomes. The prognostic performance of NLR in AL.
- Study Design. We anticipated case-control or cross-sectional articles. We did not, however, restrict our search to a specific study design.

Exclusion criteria were:

- (i) Animal, human xenograft, and cell line studies; (ii) review papers, case series, or case reports; (iii) duplicate publications.

The papers that the search strategy turned up were all given a thorough independent evaluation by two reviewers. Disagreements were settled by consensus. After excluding duplicate and clearly irrelevant articles, the full text of all potentially relevant publications was obtained and assessed for eligibility after duplicate articles. Any unclear or missing information was clarified by reaching out to the corresponding author.

### Data extraction

Two authors separately utilized predesigned abstraction forms to collect data. Disagreements were resolved by consensus. The following information was extracted from the included study: the first author’s name, the year of publication, the ethnicity of the participants (East Asian or White), the study’s location, the type of surgery (colorectal, esophageal, or gastric), the study design (prospective or retrospective), AL severity, mean age, gender, mean, mean follow up time, AL diagnosis criteria, AL diagnosis time, severity classification, AL management, exclusion criteria and comorbidities, perioperative therapies, tumor staging time of blood collection, the number of cases and controls, and the participants’ NLR levels.

### Quality assessment

The methodological quality of included studies was assessed and scored using the Newcastle-Ottawa Quality Assessment Scale (NOS), which has three parts comparability, outcome, and selection [13]. Studies of high quality received a score of six or above. In addition, Risk of bias assessment was conducted based on Cochrane-endorsed ROBINS-I assessment tool.

### Data synthesis and statistical analyses

The meta-analysis was carried out using Stata 11.2 software (Stata Corp, College Station, TX). To account for variations in NLR measuring procedures between studies, the standard mean difference (SMD) with 95% CI was employed instead of the weighted mean difference (WMD). Based on the study design, the kind of surgery, and ethnicity, subgroup analyses were also carried out. We used a random-effects model in this meta-analysis because of the significant heterogeneity between included articles. Cochran’s Q test and I^2^ statistics were used to evaluate the statistical heterogeneity: I^2^>75% and *p* value of Q test < 0.05 were considered as significant heterogeneity of results. To estimate mean and SD from median and interquartile range and/or range, we used the technique developed by Wan et al. [14]. By utilizing Egger’s test and a visual examination of the funnel plot, publication bias was determined. All statistical tests were two-sided, and statistical significance was defined as *p* < 0.05. Sensitivity analysis, subgroup analysis, and meta-regression were performed to find the source of heterogeneity. Subgroup meta-analyses was conducted based on type of surgery, study design, and ethnicity, and meta-regression was performed based on age, gender, NOS score, AL diagnosis time, and BMI. We also conducted sensitivity analysis to explore the impact of each included study on the final result.

## Results

### Identification of relevant studies

**Fig. 1:**
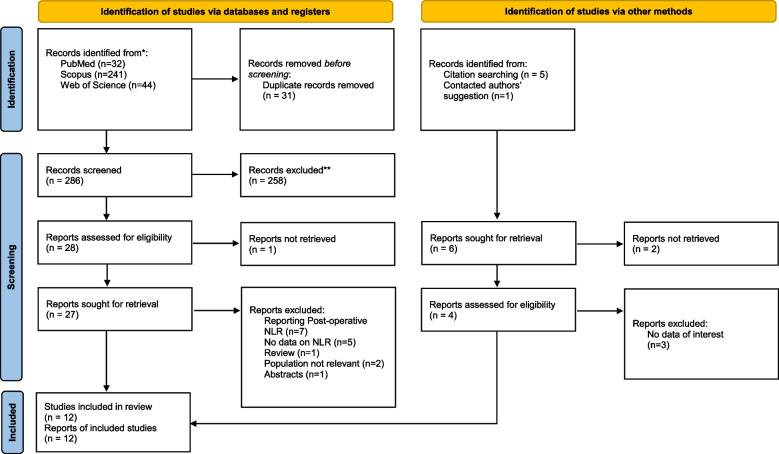
PRISMA 2020 flow diagram for new systematic reviews which includes searches of databases, registers and other sources

The database search and the manual search of the article citation list resulted in a total of 323 results. We found no further relevant studies in grey literature and hand-searching. After excluding irrelevant studies and duplicates, we included 12 studies in the present systematic review and meta-analysis for a total of 2940 patients undergoing GI surgeries, of whom 353 developed AL [4, 15–25]. The PRISMA flow diagram in Fig. 1 describes the exclusion and inclusion procedure in detail.

### Study characteristics and quality assessment

In terms of study design, there were two prospective and 10 retrospective studies. Studies were conducted in China(*n* = 3) [17, 18, 25], Italy (*n* = 2) [19, 22], Romania(n = 2) [4, 20], Japan(n = 2) [23, 24], Indonesia(*n* = 1) [15], Turkey(n = 1) [16], and Mexico(n = 1) [21]. So, there were six studies on white people, with 2106 patients, of whom 200 developed AL [4, 16, 19–22], and six studies on East Asian people, with 834 patients, of whom 153 developed AL [15, 17, 18, 23–25]. Four studies, with 513 patients, of whom 67 developed AL, included patients undergoing gastric surgery [4, 16, 21, 22], four studies, with 1783 patients, of whom 168 developed AL, included patients undergoing colorectal surgery [15, 19, 21, 24], and four studies, including 644 patients, of whom 118 developed AL, were on esophageal surgery [17, 18, 23, 25]. All of them were written in English. The quality of the studies was high, with scores ranging from 6 to 8. The general characteristics and details of the patients in each study are listed in Tables 1 and 2, respectively. In addition, Tables 3 and 4 shows the detail data from quality assessment and risk of bias assessment.

**Table 1:** General characteristics of included studies

Author	Country	Design	Type of surgery	Ethnicity	Patients	Time of blood collection	anastomotic leak				NOS score
							Yes		No		
							N	NLR	N	NLR	
Cikot, M 2018 [16]	Turkey	P	Gastric	White people	Patients undergoing elective anastomosis because of benign or malignant underlying gastrointestinal etiologies	12 hours before surgery	27	1.93 ± 0.88	73	1.85 ± 1.00	7
Palioginnis, P 2020 [19]	Italy	R	Colorectal	White people	Patients with colorectal cancer undergoing elective surgical procedure with an open or laparoscopic approach	Pre-operatively	106	3.23 ± 1.39	1326	2.96 ± 1.33	8
Radulescu, D 2020 [4]	Romania	R	Gastric	White people	Patients with gastric cancer undergoing surgery	At the moment of admission to hospital	16	5.83 ± 1.68	188	2.44 ± 0.51	6
Radulescu, D 2020 [20]	Romania	R	Colorectal	White people	Patients undergoing surgery for colon and rectal cancer	Pre-operatively before any hydroelectrolytic or protein balancing treatment	27	5.10 ± 2.02	134	2.27 ± 0.60	7
Romano, L 2020 [22]	Italy	P	Gastric	White people	Patients who underwent laparoscopic sleeve gastrectomy	The day before the operation	6	1.74 ± 0.09	145	1.84 ± 0.20	7
Sato, Sh 2020 [23]	Japan	R	Esophageal	East Asian	Patients with esophageal cancer undergoing esophagectomy with retrosternal reconstruction of the gastric conduit	Pre-operatively	38	3.23 ± 4.16	210	5.38 ± 9.06	8
Suzuki, N 2020 [24]	Japan	R	Colorectal	East Asian	Patients with rectal cancer undergoing low anterior resection	Pre-operatively	21	2.90 ± 0.79	115	2.46 ± 1.12	7
Yuliandar, AA 2020 [11]	Indonesia	R	Colorectal	East Asian	Colorectal cancer patients undergoing elective surgery	1 day before surgery	14	8.60 ± 7.86	40	5.17 ± 8.56	6
Huang, JX 2021 [17]	China	R	Esophageal	East Asian	Children with congenital esophageal atresia who underwent one-stage anastomosis in our hospital	Pre-operatively	28	3.13 ± 1.89	79	3.30 ± 1.88	7
Li, Sh. 2021 [18]	China	R	Esophageal	East Asian	Patients with esophageal cancer undergoing video-assisted esophageal resection	Pre-operatively	27	6.05 ± 9.95	64	2.98 ± 2.26	7
Rodriguez, Q 2021 [21]	Mexico	R	Gastric	White people	Patients with gastric cancer undergoing total gastrectomy	At admission 1 day previous to the operation	18	6.75 ± 5.65	40	5.05 ± 3.23	6
Wu, CX 2021 [25]	China	R	Esophageal	East Asian	Patients with esophageal cancer undergoing radical esophagectomy	Pre-operatively	25	4.44 ± 4.74	173	4.15 ± 6.60	6

*NLR* Neutrophil to lymphocyte ratio, *N *Number, *NOS *The Newcastle-Ottawa quality assessment scale, *R *Retrospective, *P *Prospective

**Table 2:** Details of included studies

First author	age	Gender (Male%)	BMI	mean follow up	AL diagnosis criteria	AL diagnosis time	severity classification	AL management	Exclusion criteria and comorbidities	perioperative therapies	AL severity (Stage III/IV vs I/II)	Tumor staging
Cikot	49	42	ND	5 days after surgery (3 month follow up for patients with AL)	AL diagnosis was based on rectal or oral contrast-induced tomography, laboratory results, and patients’ clinical findings.	5 days after surgery	Clavien- Dindo classification	Metronidazole and ceftriaxone after operation	Patients with systemic or local infection were excluded.	**Preoperatively**: enoxaparin and ceftriaxone **Postoperatively**: Enoxaparin, hydrochloride, ranitidine, hydration, and tenoxicam	IIIa: 30% IIIb: 15% IVa: 22% IVb:7% V: 26%	ND
Paliogiannis	65.8	57	25.1	within 30 days from surgery	Imaging or endoscopic techniques	within 30 days from surgery	Clavien-Dindo classification	1) Surgical operation (72% of cases) 2) Without surgical approach	Patients under the age of 18, those who had emergency surgery, and those without an anastomosis were excluded.	ND	ND	AJCC staging: 0: 1% I: 17% II: 33% III: 37% IV:12% Grading: 1:13.5% 2:71.5% 3: 15%
Radulescu1	67.75	61.8	ND	ND	ND	ND	ND	ND	Exclusion criteria: 1) Patients with autoimmune diseases and systemic inflammation 2) Those who stopped their oncological regimen 3) Those on long-term systemic anti-inflammatory medication 4) Those with concurrent cancers and secondary disseminations 5) Patients who had emergency surgery	**Preoperative:** 1) protein or electrolytic equilibration 2) Rehydration 3) chemotherapy, radiotherapy, or both	ND	Stage: I: 12% II: 32% III: 46% IV: 10%
Radulescu2	68.19	64.59	ND	ND	ND	ND	ND	ND	Exclusion criteria: Metastases, concurrent cancer, long-term anti-inflammatory medication, autoimmune disorders, incomplete oncological treatment, and systemic inflammation.	Preoperative:1) Hydroelectrolytic or protein balancing treatment; 2) Chemotherapy, radiotherapy or combined treatment	ND	Stage:I: 10.5% II: 34% III:43.5% IV: 12%
Romano	45	36.42%	40.48	5.12	1) Radiography 2) Computed tomography scan	7.16 days after surgery	Dindo-Clavien classification	1) Double pigtail drainage insertion (endoscopically) 2) Drainage (radiologically) 3) Laparoscopic surgical toilette	Participants had comorbidities like Obstructive sleep apnea, Hypertension, type 2 DM, and Dyslipidemia.	Prior surgery, a multidisciplinary team consisting of a nutritionist, psychologist, gastroenterologist, and endocrinologist assessed each patient and monitored them for a minimum of 6 months.	ND	ND
Sato	69.75	88	21.68	7 days after surgery	1) Radiographic contrast study 2) CT 3) clinical findings Formation of pus, luminal contents, or saliva via wound site accompanied with fever, local inflammation, and leukocytosis were considered indicators of a clinical leak. Contrast extravasation while the contrast study was classified as a radiologic leak. According to the radiologist, a leak in CT was described as the emergence of effusion and free gas at the anastomosis site.	6.6 days postoperatively	Esophageal Complications Consensus Group definitions AND Clavien-Dindo classification	1) Monitoring without food intake orally followed 1 week later by a second contrast exam to demonstrate healing 2) Drainage from the neck under local anesthesia 3) Antibiotic therapy 4) Mediastinal drainage under general anesthesia.	Pts had comorbidities including DM, hypertension, and COPD. Exclusion criteria: 1) Pts who underwent laryngopharyngectomy, esophagectomy, salvage esophagectomy, esophagectomy without reconstruction, reconstruction from the posterior mediastinal route, synchronous colonic or liver cancer surgery reconstruction using colon or jejunum as a conduit 2) Pts who died within 5 days after surgery due to bleeding, cardiovascular events, or unknown causes.	1) Endoscopic submucosal dissection 2) Chemoradiotherapy 3) Chemotherapy	ND	IA-IIB: 55% IIIA-IV: 45%
Suzuki	68	59.6%	21.4	44(14–89)	AL was diagnosed based on the following clinical signs: pus or stool discharge from the abdomen drain, peritonitis with tachycardia, high fever, tenderness, abdominal pain, or severe inflammation. If an abscess, free air, or fluid collection was detected around the anastomotic site, CT was used to determine the existence of AL. Asymptomatic AL, on the other hand, was difficult to evaluate since contrast enemas were not commonly performed.	ND	ND	1) re-operation 2) reconstruction of a covering stoma, and performed intraabdominal lavage and drainage 3) drainage with an abdominal drain.	Pts who had surgery utilizing alternative procedures (Hartmann’s Operation, transanal operation, Miles’ operation, total pelvic extirpation, and others) were excluded.	In complicated situations, such as tumors with extramural invasion or bulky tumors, preoperative chemotherapy or chemoradiotherapy was provided. As part of the standard preoperative evaluation for rectal surgery, a pelvic CT scan was conducted on all patients.	ND	UICC-stage: 0: 1% I: 45% II: 12% III: 29% IV: 13%
Yuliandar	53.37	57%	ND	ND	During re-surgery.	ND	ND	ND	Exclusion criteria: 1) Surgery without anastomosis (palliative stoma, Hartmann procedure, by-pass anastomosis) 2) Primary anastomosis with a protective stoma	ND	ND	ND
Huang	Neonates	69	ND	ND	ND	ND	ND	ND	Participants had comorbidities including respiratory, genitourinary, gastrointestinal, musculoskeletal, and congenital cardiovascular anomalies. Nonetheless, individuals who were unable to undergo delayed anastomosis during the first phase of the operation were excluded.	ND	ND	ND
Li	60.37	91	21.92	35.26	Fluoroscopic esophagography	7 to 14 days after surgery	ND	ND	Participants had comorbidities including Smoking, DM, alcohol consumption, and hypertension. However, patients whose diagnosis was malignant melanoma, adenocarcinoma, gastrointestinal stromal tumor, small cell carcinoma, and malignant neuroendocrine tumor; and individuals who did not get an abdominal CT scan 2 weeks prior to surgery; and patients with proven metastases prior to surgery were excluded.	ND	ND	T stage: T1-T2: 42% T3-T4: 58%
Rodriguez	61.5	37.9	ND	17.43	1) clinical or imaging indications of luminal leakage around the EJ 2) Inclusion of intestinal saliva or contents in perianastomotic drains, 3) presence of extraluminal contrast material in imaging investigations or fluid collections close to the EJ in CT scan or ultrasound.	within 1 week of operation	ND	supportive treatment including: 1) intravenous hydration 2) nothing by mouth 3) antibiotics 4) fluid collection drainage with interventional techniques	Participants had comorbidities including hypothyroidism, dyslipidemia, type 2 diabetes, and Hypertension.	1) total parenteral nutrition 2) Early feeding as tolerated	ND	ND
Wu	61.3	92.4%	ND	ND	ND	Within 30 days from surgery	ND	ND	Participants had comorbidities including DM, hypertension, and Lung disease. Patients with the following criteria were excluded: 1) Older than 80 and younger than 18 2) Active hepatic, cardiac, or renal disease 3) Chronic infection, distant metastasis, and concurrent malignancy 4) Received radiotherapy or neoadjuvant chemotherapy 5) People who underwent emergency surgery or admitted to intensive care unit after surgery 6) Those who had difficulty expelling sputum following surgery	ND	ND	AJCC stage: I: 6% II: 35% III: 56% IV: 3%

*Abbreviations:* *BMI* Body mass index, *AL* Anastomosis leak, *ND* Not declared, *DM* Diabetes mellitus, *Pt* Patient, *UICC* Union for International Cancer Control, *AJCC* American Joint Committee on Cancer, *EJ* Esophagojejunal

**Table 3:** Detail data from Quality assessment

NOS section Author	Selection				Comparability	Exposure			Total stars
	Is the case definition adequate?	Representativeness of the cases	Selection of Controls	Definition of Controls	Comparability of cases and controls	Ascertainment of exposure	Same method of ascertainment for cases and controls	Non-Response Rate	
Cikot	★	★	★	★	★	●	★	★	7
Paliogiannis	★	★	★	●	★★	★	★	★	8
Radulescu1	★	★	★	●	★	●	★	★	6
Radulescu2	★	★	★	●	★	★	★	★	7
Romano	★	●	★	★	★	★	★	★	7
Sato	★	★	★	★	★	★	★	★	8
Suzuki	★	★	★	★	★	●	★	★	7
Yuliandar	★	★	★	●	★	●	★	★	6
Huang	★	●	★	★	★	★	★	★	7
Li	★	★	★	●	★★	●	★	★	7
Rodriguez	★	★	★	●	★	●	★	★	6
Wu	★	★	★	●	★	●	★	★	6

**Table 4:** Risk of bias assessment based on Cochrane-endorsed ROBINS-I assessment tool

	Confounding	Bias in selection of participants into the study	Bias in classification of the exposure	Bias due to departures from intended exposures	Bias due to missing data	Bias in measurement of the outcome	Bias in selection of the reported results	Total
Cikot	Moderate	Low	Low	Low	Low	Low	Low	Moderate
Paliogiannis	Low	Low	Low	Low	Low	Low	Low	Moderate
Radulescu1	Moderate	Low	Low	Low	Low	Low	Low	Moderate
Radulescu2	Moderate	Low	Low	Low	Low	Low	Low	Moderate
Romano	Low	Low	Low	Low	Low	Low	Low	Moderate
Sato	Low	Low	Low	Low	Low	Low	Low	Moderate
Suzuki	Low	Low	Low	Low	Low	Low	Low	Moderate
Yuliandar	Low	Low	Low	Low	Low	Low	Low	Moderate
Huang	Moderate	Low	Low	Low	Low	Low	Low	Moderate
Li	Low	Low	Low	Low	Low	Low	Low	Moderate
Rodriguez	Low	Low	Low	Low	Low	Low	Low	Moderate
Wu	Moderate	Low	Low	Low	Low	Low	Low	Moderate

### Comparison of NLR between patients undergoing GI surgeries who developed AL and those who did not

**Fig. 2:**
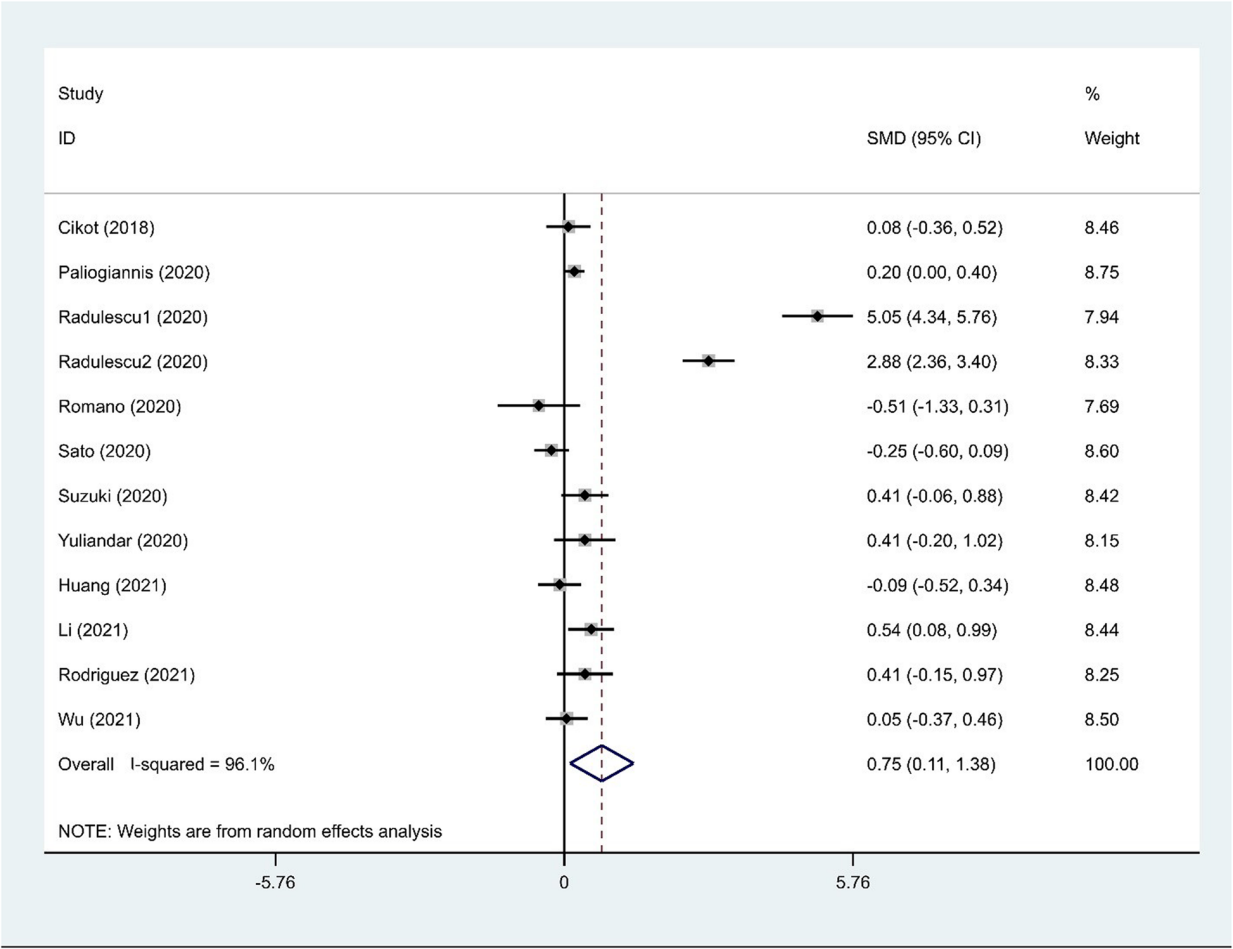
Meta-analysis of differences in NLR level between patients undergoing GI surgeries who developed AL and those who did not

After polling the data of 12 studies [4, 15–25], we found that NLR levels were significantly higher in patients undergoing GI surgeries who developed AL than those who did not (random-effects model: SMD = 0.75, 95% CI = 0.11–1.38, *p* = 0.02). We used a random effect model in our meta-analysis, because a significant heterogeneity was observed across the studies (I^2^ = 96.1%, *p* < 0.01; Fig. 2). Similar to the previous result, NLR levels were significantly higher in patients undergoing GI surgeries who developed AL than those who did not after excluding these studies (Fixed-effects model: SMD = 0.14, 95% CI = 0.02–0.26, *p* = 0.02). It shows that the presence of statistical heterogeneity is attributed to these studies.

**Fig. 3:**
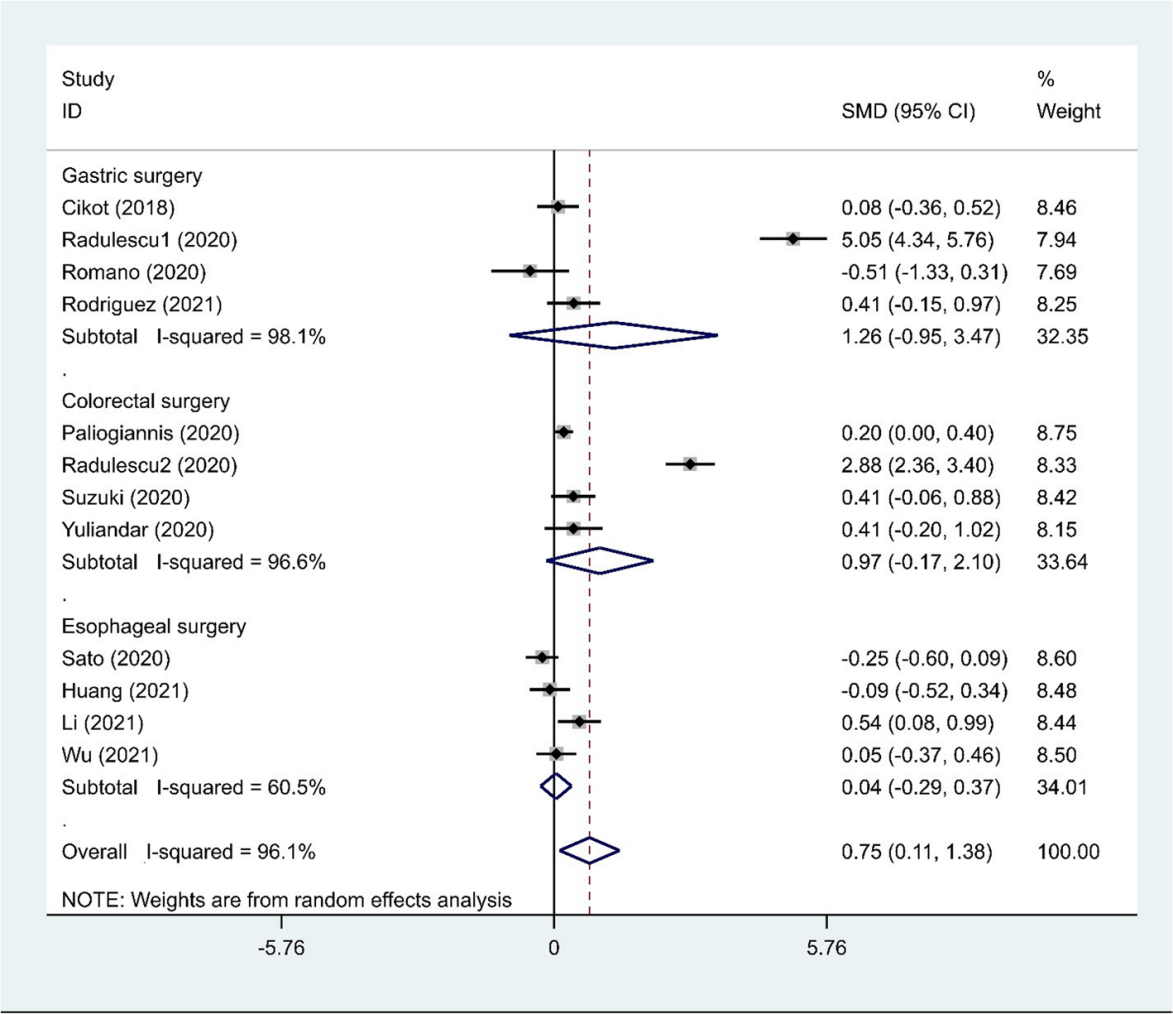
Subgroup analysis of differences in NLR level between patients undergoing GI surgeries who developed AL and those who did not, according to the type of surgery

In the subgroup analysis according to the type of surgery, we did not find any differences in NLR levels between cases and controls (SMD = 1.26, 95% CI = − 0.95–3.47, *p* = 0.26 in gastric surgery; SMD = 0.97, 95% CI = − 0.17–2.10, *p* = 0.09 in colorectal surgery; and SMD = 0.04, 95% CI = − 0.29–0.37, *p* = 0.80 in esophageal surgery) (Fig. 3).

**Fig. 4:**
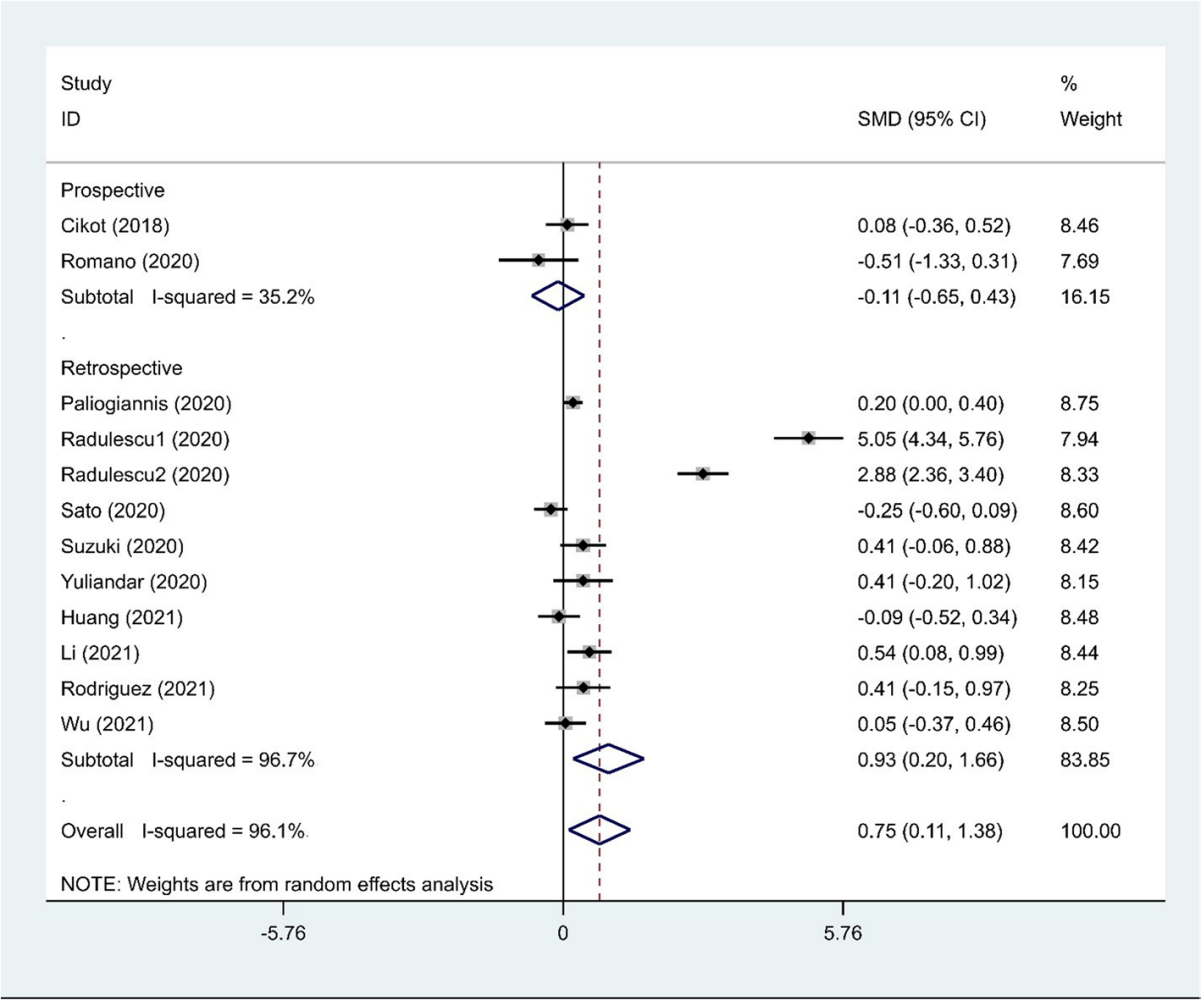
Subgroup analysis of differences in NLR level between patients undergoing GI surgeries who developed AL and those who did not, according to study design

As seen in Fig. 4**,** in the subgroup analysis according to study design, NLR levels were significantly higher in patients with AL than control group in retrospective studies (SMD = 0.93, 95% CI = 0.20–1.66, *p* = 0.01**)** but not in prospective studies (SMD = − 0.11, 95% CI = − 0.65–0.43, *p* = 0.69).

**Fig. 5:**
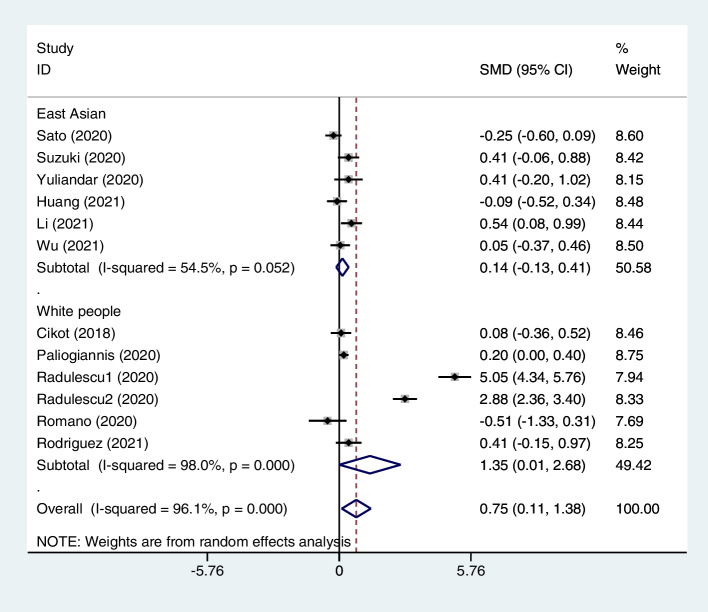
Subgroup analysis of differences in NLR level between patients undergoing GI surgeries who developed AL and those who did not, according to ethnicity

Figure 5 shows the subgroup analysis according to ethnicity. We found that NLR levels were significantly higher in patients with AL than control group in white people group (SMD = 1.35, 95% CI = 0.01–2.68, *p* = 0.04) but not in East Asian group (SMD = 0.14, 95% CI = -0.13–0.41, *p* = 0.29).

### Source of heterogeneity

The type of surgery, study design, and ethnicity cannot be the source of heterogeneity, because the subgroup analysis based on them did not reduce the heterogeneity.

In the meta-regression analysis, there was no significant effect of the mean age of cases (B = 0.08, R^2^ = 9.95, *p* = 0.18) and percentage of male patients (B = 0.0005, R^2^ = -10.64, *p* = 0.98), NOS score (B = − 0.76, R^2^ = 3.40, *p* = 0.26), AL diagnosis time (B = 0.006, R^2^ = − 40.21, *p* = 0.53), and BMI (B = − 0.03, R^2^ = 3.05, *p* = 0.29) on NLR. So they cannot be the source of heterogeneity.

### Sensitivity analysis

**Fig. 6:**
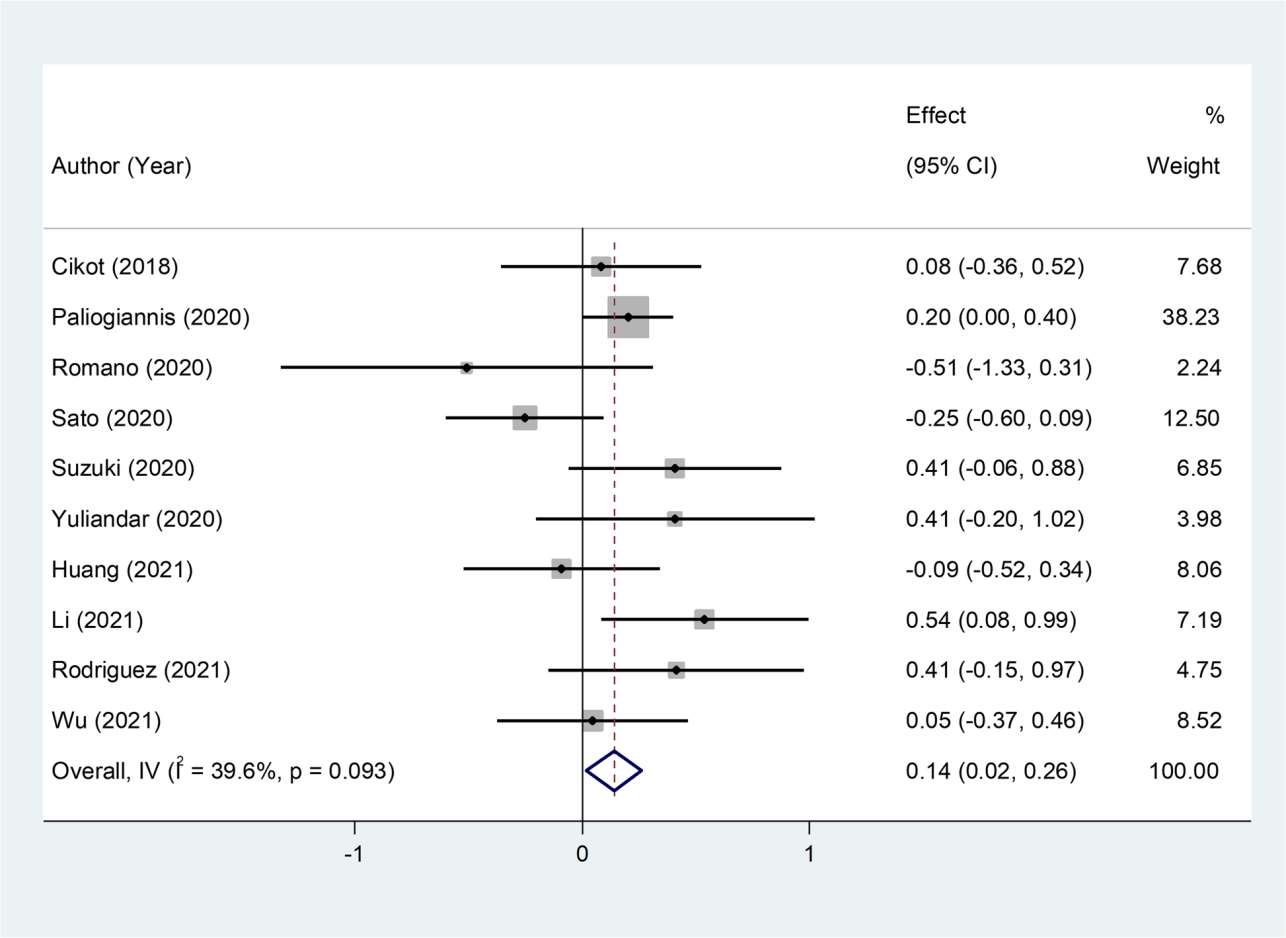
Meta-analysis of differences in NLR level between patients undergoing GI surgeries who developed AL and those who did not, without two outlying studies

However, exclusion of two outlying study from the analysis [4, 20] attenuated heterogeneity tests to non-significance (I^2^ = 39.6%, *p* = 0.093; Fig. 6); so they can be the source of heterogeneity.

### Publication bias

**Fig. 7:**
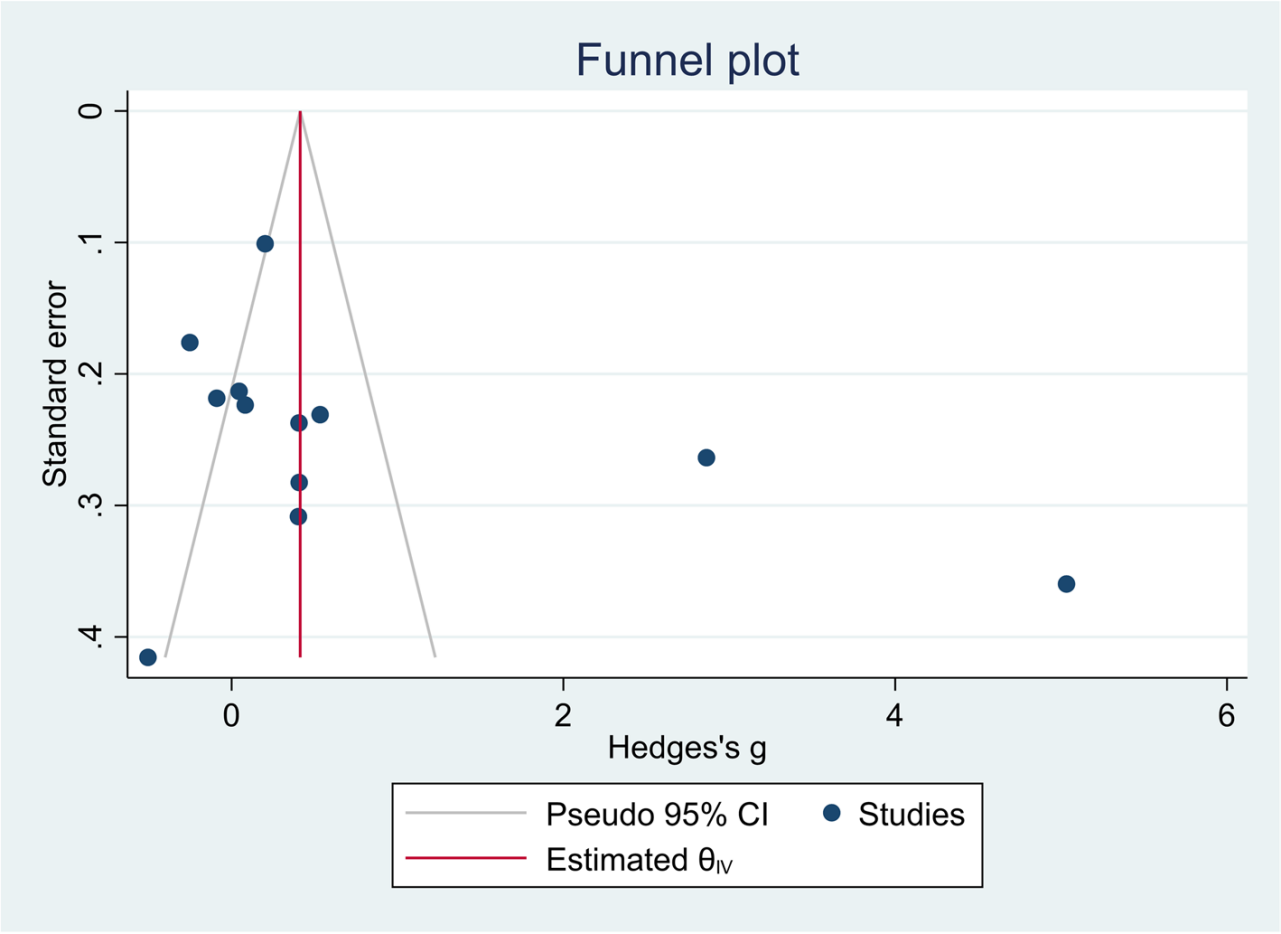
Funnel plot assessing publication bias

Figure 7 indicates no publication bias among studies on the role of NLR in AL (Egger’s test *p* = 0.23).

## Discussion

Our study found that patients with AL had significantly increased pre-operative NLR, and there is a risk increase associated with AL in patients with elevated NLR. This effect was more apparent in retrospective studies (*p* = 0.01) and among white people (*p* = 0.04). In the subgroup analysis based on surgery type, the results were insignificant; however, the pooled results of all included studies yielded significant results. This can be due to multiple reasons: 1) variability in sample sizes: The number of studies available for each surgery was limited which resulted in smaller sample size and reduced statistical power. Insufficient data within each subgroup might have led to inconclusive results. 2) Heterogeneity in patients’ populations: The lack of significance in the subgroup analysis based on surgery type could be due to variations in patients’ characteristics within each subgroup. Differences in comorbidities, disease severity, or surgical techniques may have influenced the results.

It is crucial to note the dynamic roles of neutrophils and lymphocytes in the setting of GI surgery to understand their relative proportions. In inflammatory disease, blood neutrophils often increase, and lymphocytes often decrease [4]. Neutrophils act on the ischemic areas of the anastomosis by releasing inflammatory cytokines and free radicals. In contrast, lymphocytes work to decrease inflammation and the healing process [26]. At the cellular level, the wound healing process of intestinal anastomosis follows a classical pattern: inflammation, proliferation, and remodeling. Initially, the inflammatory phase is characterized by the flood of neutrophils to the anastomotic site, and the tissue growth factor is rapidly produced. Proliferation begins as fibroblasts produce collagen in the extracellular matrix, most notably within the submucosal layer. Finally, the newly formed tissue begins to remodel [27].

Neutrophil levels increase within 1 hour and persist for the first 48 hours [28]. At the anastomotic trance, neutrophils phagocytose bacteria and foreign particles. This phagocytic activity replaces that of macrophages as their activity is decreased. Additionally, neutrophils aid in the healing process by releasing oxidants and hydrolytic enzymes [29]. Lymphocytes begin their course in the inflammatory process after the first 72 hours. Lymphocytes mediate the healing process through extracellular matrix synthesis and collagen remodeling [30].

The inflammatory stage, marked by the influx of neutrophils, persists for as long as there are bacteria and foreign particles to phagocytose within the digestive lumen. Therefore, this stage will be abnormally prolonged in patients with pre-existing proinflammatory conditions. This will consequently result in increased tissue injury and decreased extracellular matrix synthesis [31]. Ultimately, this persistent proinflammatory state will lead to an insufficient cure of anastomosis with dehiscence [4]. Morimoto and colleagues illustrated the specific role tumor-related inflammation has in wound pathology that results in AL in patients with colorectal cancer. Their research demonstrated the predictive role white blood cells have, in addition to the NLR, in AL prevention and treatment [32].

Radulescu and colleagues investigated NLR as a predictor of AL in patients undergoing gastric resection. Retrospectively, a pre-operative NLR greater than or equal to 3.54 was significantly correlated with post-operative AL. It is of note that NLR increased directly proportional to tumor stage. Therefore, patients with more severe malignancies have an increased risk of AL [4]. Forget, and colleagues found a statistically significant elevated NLR on postoperative day seven following abdominal surgery in patients with a post-operative infection versus patients with good postoperative outcomes. Their optimal NLR threshold on postoperative day 7 is 5.5, (sensitivity: 66%, specificity: 77%) [33]. While this is a statistically significant marker, the hospital course of patients is frequently less than 7 days, and their AL are often already manifested by the time they return to the hospital. In their study, Pantoja Pachjoa and colleagues did not find a statistically significant difference in NLR values between patients with or without post-operative infections until post-operative day 5 [34].

Pantoja Pachjoa and colleagues found that C-reactive protein (CRP) served as a more accurate prognostic indicator of AL than NLR in their retrospective cohort following colorectal surgery. Their data suggest that the CRP at post-operative day 5 serves the most powerful predictive role at values greater than 54 mg/dL [34]. Additionally, Ramanathan and colleagues suggested that while an open surgery approach for colon cancer resection carries a greater inflammatory response than a laparoscopic approach, the predictive thresholds of CRP in post-operative complications were similar across both approaches. CRP values greater than 180 mg/dL on day 3 and 140 mg/dL on day four served as a threshold of post-operative infections in both surgical approaches. Notably, the patients undergoing laparoscopic procedures have lower baseline CRP values pre-operatively [35]. As CRP is a measure of the inflammatory response, these patients likely had fewer inflammatory comorbidities. These previous studies suggest a relationship between a CRP threshold and the development of a post-operative infection across surgical approaches and comorbidity.

These data suggest a use for both NLR and CRP in predicting AL and post-operative infection. Further data is needed to determine under which conditions either value is useful. This includes scenarios assessing patients with various comorbidities. Additionally, developing a hospital course that may involve returning for a blood draw after discharge may prove to be cost-effective by allowing clinicians to detect the potential manifestation of a post-operative infection. This would aid in the prevention and treatment of life-threatening poor post-operative outcomes. Further research is needed to determine the exact timeline of this proposed course, as well as the cost-effectiveness and reality of follow-up.

The results indicate a difference in the predictive value of NLR in retrospective (*p* = 0.023) compared to prospective studies (*p* = 0.49). We speculate that this effect may be due to the smaller number of prospective studies compared to retrospective studies. Thus, more prospective studies may help clarify whether there is a difference between retrospective and prospective studies in the context of the NLR and its predictive value for AL.

Furthermore, we found a difference in NLR predictive value for AL when comparing studies with white people (*p* = 0.016) compared to East Asian patients (*p* = 0.995). Specifically, higher pre-operative NLR values were found in Caucasian patients with AL relative to those with normal healing, but this effect was not replicated in the East Asian group. We propose that these differences may be attributed to diet, leading to microbiome composition [36]. Furthermore, mice fed a high-fat obesogenic Western diet had an increased risk of AL, which was prevented by a short course of low-fat and high-fiber standard chow diet [37]. The microbiome also varies significantly by ethnicity, which is an important proxy for dietary and lifestyle differences between groups [38, 39]. Thus, the differences between East Asian and Caucasian individuals in our study may be attributed to dietary and lifestyle factors between ethnicities.

Not only the pre-operative NLR but also the post-operative NLR has been an object of research [34, 40–42]. Walker et al. examined 136 patients undergoing colonic and rectal anastomosis and found NLR to be a significant predictor of AL, particularly on post-operative days 3 and 4 [41]. This mirrors the findings of Liu et al. [42] and Al Lawati et al. [41], who studied 787 patients with rectal cancer and 333 patients with esophageal adenocarcinoma, respectively. Liu et al. found NLR to be a significant predictor of AL on post-operative days 3 and 5 (*P* < 0.05) [41]. Al Lawati et al. also found NLR to be a significant predictor of AL on post-operative days 1, 2, and 3 (*P* < 0.001, < 0.001, < 0.001, respectively) [41]. Furthermore, patients with AL demonstrated rising NLR trends in the early post-operative period [41]. Meanwhile, a low NLR value on post-operative day three was associated with a high negative predictive value (92.4%) for AL. In contrast to the above studies, Pantoja Pachajoa et al. included 116 patients who underwent colorectal surgery with anastomosis and found post-operative NLR not to be a significant predictor of AL [34]. Instead, CRP was the best predictor, especially on post-operative day 5 (*p* < 0.001). Elevated post-operative CRP has also been shown to be a reliable predictor of AL in other studies and sometimes superior to that of NLR [34, 40, 43–45]. However, NLR is cheap and conveniently measured, so it likely still retains utility in post-operative management following anastomosis [40].

### Limitations and strengths

There are a few issues with our research that need to be addressed. First, there was a high level of heterogeneity in the papers we included in our analysis. Although the random effect model compensated for this, such measures may not completely solve the problem of heterogeneity. High heterogeneity might be attributed to the fact that many methods were employed to evaluate NLR in selected studies, and within those utilized, there is also a risk of user variability owing to their subjective character. Furthermore, most of the included papers on this issue were retrospective. More prospective investigations are therefore suggested. Finally, we could not perform subgroup analysis based on diagnosis criteria, severity classification, management, comorbidities and perioperative therapies, because the data of included studies was incomplete and heterogeneous to the extent that we cannot categorize them in groups. However, these variables could be the possible source of heterogeneity in our study.

Nonetheless, the current review has three major strengths. To our knowledge, this is the first meta-analysis that investigates the relationship between NLR and AL. Second, the studies were only included in the final analysis if they fulfilled strict and clear inclusion and exclusion criteria. Third, our systematic search, in combination with a manual assessment of references from resulting documents, guaranteed a complete and credible literature search, which is a significant strength of our research.

### Conclusion

Our study showed that NLR level is elevated in patients with AL than those without AL. The results of our study support an association between elevated pre-operative NLR values and increased risk of AL among patients undergoing GI surgeries. NLR represents a unique inflammatory marker whose elevation in AL implicates immune system imbalance in the pathogenesis of the disease. Further, our findings support NLR as a promising biomarker that can be readily integrated into clinical settings to aid in predicting and preventing AL. Ultimately, with the development of new biomarkers and therapeutic modalities, we can better prevent and treat AL to decrease long-term morbidity and mortality.

## Data Availability

The dataset supporting the conclusions of this article is included within the article.

## Abbreviations

NLR: Neutrophil to lymphocyte ratio
SMD: Standardized mean difference
AL: Anastomotic leak
GI: Gastrointestinal
CRP: C-reactive protein
SD: Standard deviation
95% CI: 95% confidence interval
CRP: C-reactive protein
N: Number
NOS: The Newcastle-Ottawa Quality Assessment Scale
R: Retrospective
P: Prospective
PRISMA: Preferred Reporting Items for Systematic Reviews and Meta-Analyses
SROC: Summary receiver operating characteristic
DOR: Diagnostic odds ratio

## References

[1] Kumarasamy C (2019). Prognostic significance of blood inflammatory biomarkers NLR, PLR, and LMR in cancer-a protocol for systematic review and meta-analysis. Med (Baltimore)..

[2] Clemente-Gutiérrez U (2021). Usefulness of inflammatory markers in detecting esophagojejunostomy leakage. Rev Gastroenterol Mex (Engl Ed)..

[3] Tsuei BJ, Schwartz RW (2004). Management of the difficult duodenum. Curr Surg..

[4] Radulescu D, et al. Neutrophil/lymphocyte ratio as predictor of anastomotic leak after gastric Cancer surgery. Diagnostics (Basel). 2020;10(10) 10.3390/diagnostics10100799 PMC7601164 33050137

[5] Licht E (2016). Endoscopic Management of Esophageal Anastomotic Leaks after Surgery for malignant disease. Ann Thorac Surg..

[6] Kang CY (2013). Risk factors for anastomotic leakage after anterior resection for rectal cancer. JAMA Surg..

[7] Ashraf SQ (2013). The economic impact of anastomotic leakage after anterior resections in English NHS hospitals: are we adequately remunerating them?. Color Dis..

[8] Salimi M (2022). Utilization of chest tube as an esophagus stent in pediatric caustic injuries: a retrospective study. World J Clin Pediatr..

[9] Akbari-Khezrabadi A (2022). Can anthropometric indices predict the chance of hypertension? A multicentre cross-sectional study in Iran. BMJ Open..

[10] Ostovari A, Shahabinezhad A, Sarejloo S, Mesbahi SA, Saem J, Hamidianshirazi Y, Bananzadeh A, Paydar S, Salimi M. Thromboembolic Events among Multiple Trauma Victims with Pelvic Fractures with Injury Severity Score Greater Than 16 with and without Deep Vein Thrombosis Prophylactic Doses of Enoxaparin. Surgery Insights. 2023.

[11] Nikoo MH (2021). Systolic dysfunction and complete heart block as complications of fulminant myocarditis in a recovered COVID-19 patient. J Cardiol Cases..

[12] Page MJ (2021). The PRISMA 2020 statement: an updated guideline for reporting systematic reviews. Int J Surg..

[13] Wells G (2014). Newcastle-Ottawa quality assessment scale cohort studies.

[14] Wan X (2014). Estimating the sample mean and standard deviation from the sample size, median, range and/or interquartile range. BMC Med Res Methodol..

[15] Yuliandar AA, Prihantono BS (2020). Relationship of neutrophil-to-lymphocyte ratio with anastomosis leakage as complication of colorectal surgery in colorectal Cancer patients. Int J Curr Res Rev..

[16] Cikot M (2018). The importance of presepsin value in detection of gastrointestinal anastomotic leak: a pilot study. J Surg Res..

[17] Huang J-X (2021). Risk factors for anastomotic complications after one-stage anastomosis for oesophageal atresia. J Cardiothorac Surg..

[18] Li S (2022). Body composition in relation to postoperative anastomotic leakage and overall survival in patients with esophageal cancer. Nutrition..

[19] Paliogiannis P (2020). Blood cell count indexes as predictors of anastomotic leakage in elective colorectal surgery: a multicenter study on 1432 patients. World J Surg Oncol..

[20] Radulescu D, Baleanu VD, Nicolaescu A, Lazar M, Bica M, Georgescu EF, Surlin MV, Georgescu I, Popescu AT. Neutrophil/Lymphocyte Ratio after Flow Citometry Periferic Blood Cell Detection-Predictive Marker of Anastomotic Fistula in Colorectal Cancer Surgery. 2020.

[21] Rodríguez-Quintero JH (2022). Predictors of anastomotic leak after total gastrectomy in patients with adenocarcinoma. Y CIRUJANOS..

[22] Romano L (2021). Laparoscopic sleeve gastrectomy: a role of inflammatory markers in the early detection of gastric leak. J Minimal Access Surg..

[23] Sato S (2020). Size of the thoracic inlet predicts cervical anastomotic leak after retrosternal reconstruction after esophagectomy for esophageal cancer. Surgery..

[24] Suzuki N (2021). Determining the protective characteristics and risk factors for the development of anastomotic leakage after low anterior resection for rectal cancer. Surg Today..

[25] Wu C-X (2021). Peripheral blood inflammation indices are effective predictors of anastomotic leakage in elective esophageal surgery. J Gastrointest Oncol..

[26] Frangogiannis NG, Smith CW, Entman ML (2002). The inflammatory response in myocardial infarction. Cardiovasc Res..

[27] Yuan Y, Wang KN, Chen LQ (2015). Esophageal anastomosis. Dis Esophagus..

[28] Broughton G, Janis JE, Attinger CE (2006). The basic science of wound healing. Plast Reconstr Surg..

[29] Tymen SD (2013). Restraint stress alters neutrophil and macrophage phenotypes during wound healing. Brain Behav Immun..

[30] Wang WT (2018). Impaired cutaneous T-cell attracting chemokine elevation and adipose-derived stromal cell migration in a high-glucose environment cause poor diabetic wound healing. Kaohsiung J Med Sci..

[31] Nathan C (2002). Points of control in inflammation. Nature..

[32] Morimoto M (2021). Preoperative white blood cell count predicts anastomotic leakage in patients with left-sided colorectal cancer. PLoS One..

[33] Forget P, Dinant V, De Kock M (2015). Is the neutrophil-to-lymphocyte ratio more correlated than C-reactive protein with postoperative complications after major abdominal surgery?. PeerJ..

[34] Pantoja Pachajoa DA (2021). Neutrophil-to-lymphocyte ratio vs C-reactive protein as early predictors of anastomotic leakage after colorectal surgery: a retrospective cohort study. Ann Med Surg (Lond)..

[35] Ramanathan ML (2015). The impact of open versus laparoscopic resection for colon cancer on C-reactive protein concentrations as a predictor of postoperative infective complications. Ann Surg Oncol..

[36] Williamson AJ, Alverdy JC (2021). Influence of the microbiome on anastomotic leak. Clin Colon Rectal Surg..

[37] Hyoju SK (2020). Low-fat/high-fibre diet prehabilitation improves anastomotic healing via the microbiome: an experimental model. Br J Surg..

[38] Dwiyanto J (2021). Ethnicity influences the gut microbiota of individuals sharing a geographical location: a cross-sectional study from a middle-income country. Sci Rep..

[39] Ang QY, et al. The east Asian gut microbiome is distinct from colocalized white subjects and connected to metabolic health. Elife. 2021:10. 10.7554/eLife.70349 PMC8612731 34617511

[40] Walker PA, Kunjuraman B, Bartolo DC. Neutrophil‐to‐lymphocyte ratio predicts anastomotic dehiscence. ANZ journal of surgery. 2018;88(7-8):E573–7. 10.1111/ans.14369 29377500

[41] Al Lawati Y (2021). The predictive value of inflammatory biomarkers in esophageal anastomotic leaks. Ann Thorac Surg..

[42] Liu YJ (2020). The clinical values of neutrophil-to-lymphocyte ratio as an early predictor of anastomotic leak in postoperative rectal cancer patients. Zhonghua Zhong Liu Za Zhi..

[43] Baeza-Murcia M (2021). Early diagnosis of anastomotic leakage in colorectal surgery: prospective observational study of the utility of inflammatory markers and determination of pathological levels. Updat Surg..

[44] Scepanovic MS (2013). C-reactive protein as an early predictor for anastomotic leakage in elective abdominal surgery. Tech Coloproctol..

[45] Messias BA (2020). Serum C-reactive protein is a useful marker to exclude anastomotic leakage after colorectal surgery. Sci Rep..

